# Ketamine Does Not Change Natural Killer Cell Cytotoxicity in Patients Undergoing Cancer Surgery: Basic Experiment and Clinical Trial

**DOI:** 10.1155/2022/8946269

**Published:** 2022-04-08

**Authors:** Mirei Kubota, Hidetomo Niwa, Kazuhiko Seya, Jun Kawaguchi, Tetsuya Kushikata, Kazuyoshi Hirota

**Affiliations:** ^1^Department of Anesthesiology, Hirosaki University Hospital, 53 Honcho, Hirosaki, Aomori 036-8563, Japan; ^2^Department of Anesthesiology, Hirosaki University Graduate School of Medicine, 5 Zaifucho, Hirosaki, Aomori 036-8562, Japan; ^3^Department of Vascular Biology, Hirosaki University Graduate School of Medicine, 5 Zaifucho, Hirosaki, Aomori 036-8562, Japan

## Abstract

**Background:**

The natural killer cell cytotoxicity (NKCC) suppressed by nociceptive stimuli, systemic inflammation, and drugs used during cancer surgery may be associated with poor outcomes. We investigated the potential modulation of ketamine on NKCC *in vitro* and in a clinical setting during cancer surgery. *Subjects and Methods.* The NK cell line KHYG1 was cultured for the *in vitro* experiments. The NK cells were treated with 3 and 10 *μ*M ketamine (the ketamine groups) or without ketamine (the control) for 4, 24, and 48 h. The posttreatment NKCC was measured with a lactate dehydrogenase assay and compared among the treatment groups. For the clinical study, lung cancer patients (*n* = 38) and prostate cancer patients (*n* = 60) who underwent radical cancer surgeries at a teaching hospital were recruited. The patients received propofol and remifentanil superposed with or without ketamine (ketamine group, *n* = 47; control group, *n* = 51). The primary outcome was the difference in NKCC between these groups.

**Results:**

In the *in vitro* experiment, the cytotoxicity of NK cells was similar with or without ketamine at all of the incubation periods. The patients' NKCC was also not significantly different between the patients who received ketamine and those who did not, at the baseline (36.6 ± 16.7% vs. 38.5 ± 15.4%, *p* = 0.56) and at 24 h (25.6 ± 12.9% vs. 27.7 ± 13.5%, respectively, *p* = 0.49).

**Conclusion:**

Ketamine does not change NKCC *in vitro* or in the clinical setting of patients who undergo cancer surgery. This trial is registered with UMIN000021231.

## 1. Introduction

Natural killer (NK) cells are the first line of defense against cancerous, viral infected, and stressed cells [1]. As an integral part of innate immunity, NK cells stand guard over and rapidly respond to transformed and infected cells. Without presensitization by dendritic cells, NK cells kill these cells by their direct cytotoxicity and produce cytokines [2] before the adaptive immune response is underway [3]. A compelling report showed that a medium-high cytotoxic level of peripheral blood lymphocytes including NK cells was significantly associated with a low risk of cancer morbidity, indicating that NK cells play pivotal roles in anticancer immunity [4]. Unfortunately, during the resection of cancer lesions, surgical stress (i.e., the nociceptive stimuli and the systemic inflammation due to the surgical trauma) severely suppresses NK cell function [5, 6]. This profound suppression of NK cell activity persists for weeks after surgery, promotes the recurrence and metastases [6], and is linked to poor cancer surgery outcomes—as almost all cancer deaths after primary surgery are due to cancer recurrence or metastases [7].

Postsurgery anticancer immune suppression depends on the degree of surgical stress. The antinociceptive action of anesthesia is thus suspected to affect cancer surgery outcomes [8]. Regional anesthesia is expected to reduce such immunosuppression [8] since it strongly prevents nociceptive stimuli (i.e., the body's surgical stress) [9]. In contrast, most anesthetic drugs such as opioids and volatile agents can directly impair NK cell function [8, 10, 11]. Some animal studies have shown that the N-methyl-D-aspartate receptor antagonist ketamine can also directly depress NK cell function [12, 13] and worsen cancer metastases [12]. However, it has not been established whether ketamine itself suppresses NK cell function in humans.

Ketamine has long provided a high quality of analgesia against surgical trauma [14, 15], and it also regulates the excessive inflammatory response to surgical trauma [16]. We thus speculated that such an antinociceptive effect as well as an anti-inflammatory effect of ketamine could attenuate the body's response to surgical stress and impair postsurgery immunosuppression. To test this hypothesis, we conducted a randomized clinical trial [17] and evaluated the natural killer cell cytotoxicity (NKCC) in patients who were undergoing prostate cancer surgery. However, our speculation could not be answered in that trial because of an unexpected study limitation; some of the anesthetic agents used in the trial were not standardized, although we did have a standardized plan [17]. To overcome this study limitation and to clarify whether ketamine modulates the NKCC in patients undergoing cancer surgery, we conducted the present additional clinical trial and analyzed the combined data of the present and previous trials with a sufficient number of patients. We also conducted an *in vitro* experiment to test whether ketamine directly suppresses NKCC.

## 2. Methods

### 2.1. Basic Approach

#### 2.1.1. Cell Culture

We obtained the cell line K562 (which are human chronic myelogenous leukemia cells that are sensitive to NK cells) and the cell line KHYG1 (human NK leukemia cells) from Japan's National Institutes of Biomedical Innovation, Health and Nutrition and the JCRB (Japanese Collection of Research Bioresources Cell Bank, Osaka, Japan), respectively. The K562 and KHYG1 cells were plated on 75 cm^2^ tissue culture flasks. The K562 cells were cultured in RPMI 1640 medium (cat.# R8758, Sigma-Aldrich, St. Louis, MO, USA) that we supplemented with 10% fetal bovine serum (FBS; cat.# 176012, Sigma-Aldrich). We cultured KHYG1 cells in RPMI 1640 with 10% FBS plus 100 U/ml of recombinant human interleukin- (rhIL-) 2 (cat.# 11147528001, Roche Applied Science, Mannheim, Germany). A humidified atmosphere of 95% air and 5% CO_2_ was used to grow both cell lines at 37°C, and the cells were harvested from the flasks for further passages.

#### 2.1.2. Ketamine Treatment and Lactate Dehydrogenase (LDH) Cytotoxicity Assay

KHYG1 cells were seeded in 100 mm plates (2 × 10^5^ cells/ml) and incubated for 4, 24, and 48 h in RPMI 1640 plus 10% FBS and 100 U/ml of rhIL-2 containing the indicated concentrations of ketamine (0, 3, and 10 *μ*M). Both 3 *μ*M and 10 *μ*M ketamine are considered the plasma concentrations that are necessary to maintain anesthesia in a clinical setting [16].

After each duration of ketamine treatment, we collected KHYG1 cells by centrifugation (1000 rpm at 20°C, 10 min), resuspended them in RPMI 1640 with 1% FBS, and seeded them in 96-well plates (50 × 10^3^ or 25 × 10^3^ cells/well). We also collected K562 cells by centrifugation (1000 rpm at 20°C, 10 min), resuspended them in RPMI 1640 with 1% FBS, and seeded them in the same plates as KHYG1 (2.5 × 10^3^ cells/well). The two types of cells were thus mixed at an effector-to-target cell ratio of 20 : 1 or 10 : 1.

Next, the KHYG1 and K562 cells were cocultured for 4 h at 37°C in a humidified incubator with 95% air and 5% CO_2_. After the coculture, the NKCC was determined by an assay that measures the release of lactate dehydrogenase (LDH) from K562 cells, i.e., the Cytotoxicity Detection Kit Plus (LDH) (cat.# 4744934, Sigma Aldrich) in accord with the manufacturer's protocol. The measurement of the NKCC was performed in triplicate. This LDH cytotoxicity assay is a colorimetric assay that is a reliable method for determining cellular cytotoxicity and is reported to precisely identify the values of NKCC [18, 19]. KHYG1 cells treated with 10 *μ*M prednisolone were used to detect the reduction of NKCC as the positive control.

### 2.2. Clinical Study: The Additional Randomized Controlled Clinical Trial

To clarify the clinical outcomes of ketamine treatment with a sufficient number of patients as well as to overcome our previous study limitation (i.e., some of the anesthetic agents used in the previous trial were not standardized), we conducted an additional clinical trial and analyzed the combined clinical data of our previous trial (registration no. UMIN000021231, ) and the present clinical trial. The previous trial was conducted in patients who had each undergone a minimally invasive prostatectomy; they were randomly allocated to receive general anesthesia superposed with ketamine (ketamine group) or general anesthesia without ketamine (control group) according to the same protocol and with the same outcome measures as those in the present clinical trial of patients undergoing lung cancer surgery.

#### 2.2.1. Patient Selection

This prospective, randomized controlled trial was conducted at the Hirosaki University Hospital (a teaching hospital). Before the patients' registration, this trial was registered at the University Hospital Medical Information Network (registration no. UMIN000021231, principal investigator: H. Niwa, registration date: Feb. 28, 2016).

We used a computer-generated table to randomly assign patients who underwent a thoracoscopy-assisted lung lobectomy to receive general anesthesia superposed with ketamine (ketamine group, *n* = 23) or without ketamine (the control group, *n* = 26). We conducted a block randomization with two sets of blocks of two random combinations (ketamine and no ketamine treatment). The random allocation sequence was generated by one investigator (H.N.). The patients' enrolment and allocation to interventions were done by other investigators (M.K. and J.K.). Patients aged ≥18 years whose American Society of Anesthesiologists (ASA) physical status was I, II, or III were candidates for inclusion in this study and were approached for consent. Urgent cases and patients who had an acute medical disease, a cognitive disorder, or a history of other cancer treatment within the prior 12 months were excluded. The patients and the investigators who measured the patients' laboratory data were blinded in this study. The attending anesthesiologists were not blinded after the patients were assigned to interventions.

The study was approved by the Institutional Review Board of the Hirosaki University Graduate School of Medicine (approval no. 2015-205, approval date January 19, 2016; rinri@hirosaki-u.ac.jp). All patients provided written informed consent for their participation.

#### 2.2.2. Anesthesia

The present patients' anesthesia was conducted in accord with our previous study protocol [17]. Briefly, the anesthetic agents and techniques were standardized as follows. The ketamine group patients received propofol and remifentanil with ketamine. The control group patients received propofol and remifentanil without ketamine. Considering the ethical concerns, a placebo was not used in the control group. Thoracic epidural catheters were placed at T4/5, 5/6, and 6/7 in all patients before surgery. The successful placement was then confirmed by a “cold test.” Anesthetic induction was performed using a combination of propofol (0.5–1.0 mg kg^−1^), remifentanil (0.1–0.5 *μ*g kg^−1^ min^−1^), and rocuronium (0.6 mg kg^−1^) with/without ketamine (1 mg kg^−1^). For the maintenance of anesthesia, propofol (3–7 mg kg^−1^ hr^−1^) and remifentanil (0.05–0.5 *μ*g kg^−1^ min^−1^) with/without ketamine (0.3 mg kg^−1^ hr^−1^) were administered and titrated to maintain the patient's hemodynamics in a clinically acceptable range with *electroencephalography (EEG) guidance* using a target bispectral index (BIS) value of approx. 40–60 (BIS-XP® system, Aspect Medical Systems, Leiden, The Netherlands). At the discretion of the attending anesthesiologists, 0.2 mg kg^−1^ of rocuronium was added during surgery in both groups.

After the induction of general anesthesia, 2 mg of morphine was given epidurally followed by a continuous injection of 0.167%–0.25% levobupivacaine at 4 ml/h in both groups. Standard monitoring such as ECG, BIS, and pulse oximetry was used after the patient was brought to the operating room. Before the induction of anesthesia, direct arterial blood pressure was measured using a radial artery catheter.

#### 2.2.3. Postoperative Pain Management

When the surgeon began to close the chest, patients in both groups were given 10–20 mg kg^−1^ of intravenous acetaminophen. At the same time, the continuous administration of ketamine was stopped in the ketamine group. After the operation, patient-controlled epidural analgesia was provided to the patients transferred to the ICU as 8 mg of morphine in 100 ml of 0.25% levobupivacaine, administered epidurally at 2 ml/h (a 2 ml bolus and a lockout time of 30 min). A 10 mg ^−1^ dose of acetaminophen was administered intravenously every 6 h postsurgery.

#### 2.2.4. Data Measurements

The patients' age, height, body weight, and ASA physical status classification were determined before surgery. The following surgical data of each patient were recorded after surgery: the total dose of each anesthetic administered, the values on a numerical rating scale (NRS) used to evaluate the pain intensity after surgery, and the duration of surgery/anesthesia.

#### 2.2.5. The Measurement of the NKCC

Blood sampling was conducted before the anesthesia was initiated (baseline) and at 24 h after the induction of anesthesia. Each patient's NKCC was measured with a chromium-51 (^51^Cr) release assay in duplicate, according to the manufacturer's protocol (SRL, Tokyo). Briefly, peripheral blood mononuclear cells (PBMCs) (monocytes and lymphocytes) and ^51^Cr-labeled K562 cells were cocultured for 3.5 h at an effector-to-target ratio of 20 : 1. The label was thus released from K562 cells and counted using a gamma counter. The NKCC was calculated according to the following formula: NKCC (%) = (experimental release [counts per minute, cpm] − spontaneous release [cpm]) (max.release [cpm] − spontaneous release [cpm])^−1^ × 100.

#### 2.2.6. The Neutrophil-Lymphocyte Ratio (NLR) and the Platelet-Lymphocyte Ratio (PLR)

The patients' pre- and postoperative neutrophil-lymphocyte ratios (NLR) (neutrophil lymphocyte count^−1^) and platelet-lymphocyte ratios (PLR) (platelet lymphocyte count^−1^) were calculated and compared. The pre- and postoperative data were collected on the last day before the patient's admission and within 48 h after the induction of anesthesia, respectively.

#### 2.2.7. The Measurement of Serum IL-6

Blood sampling for the measurement of interleukin- (IL-) 6 was performed before anesthesia was initiated (baseline), at 6 h, and at 24 h after the induction of anesthesia. The IL-6 was determined by a chemiluminescent enzyme immunoassay (CLEIA) using the Human IL-6 CLEIA kit (Fujirebio, Tokyo) according to the manufacturer's protocol. Briefly, a blood sample was taken at each time point, allowed to clot at room temperature, and then centrifuged at 1000 rpm for 10 min at 4°C. The serum was collected and quickly frozen at −20°C and stored until the day of analysis.

#### 2.2.8. Outcome Measures of the Clinical Trial

The primary outcome of the clinical trial was the difference in NKCC between the ketamine and control groups. The secondary outcomes were the between-group differences in the NLR, PLR, and IL-6 level.

### 2.3. Statistical Analyses

We determined the mean ± standard deviation (SD) for continuous variables with a normal distribution. The medians (interquartile range) are presented for variables that were not normally distributed. Probability (*p*) values < 0.05 were accepted as significant. We used the *χ*^2^-test for the analysis of categorical data. An independent sample *t*-test and one- or two-way analysis of variance (ANOVA) with Tukey's honestly significant difference test or a Bonferroni correction were used for continuous variables with normal distributions. The Mann-Whitney rank-sum test was used for continuous variables with a nonnormal distribution. A repeated-measures ANOVA with a Bonferroni correction was conducted to determine the significance of differences in NKCC and IL-6 values between the ketamine and control groups.

*A priori* sample size calculations for the analysis using the combined data of our previous trial and that of the present trial were done using G∗Power 3 software [20]. A power analysis was performed by using a repeated-measures ANOVA with an effect size of 0.25 (number of groups: 2 and number of measures: 2). A total of 98 patients were needed to detect a difference in NKCC for the power of 0.80 at a two-sided alpha level of 0.05. All statistical analyses were conducted with IBM SPSS® statistics ver. 22.0 software (IBM, Tokyo).

## 3. Results

### 3.1. Basic Experiment

**Figure 1:**
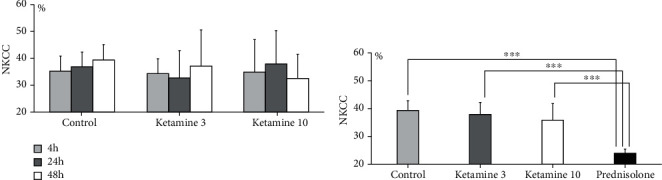
The natural killer cytotoxicity (NKCC) in the basic experiment. (a) The NKCC at an effector-to-target cell ratio of 20 : 1. KHYG1 cells were incubated for 4, 24, and 48 h with the indicated concentrations of ketamine (0, 3, and 10 *μ*M), collected by centrifugation, resuspended, and seeded in 96-well plates (50 × 10^3^ cells/well). K562 cells (2.5 × 10^3^ cells/well) were seeded in the same plates as the KHYG1 cells. The two types of cells were thus mixed at an effector-to-target cell ratio of 20 : 1. After 4 h of coculture, we determined the NKCC by measuring the lactate dehydrogenase (LDH) release from K562 cells according to the assay manufacturer's protocol. No significant difference in NKCC was revealed between the control and ketamine groups at any time point. (b) KHYG1 cells were incubated for 24 h with the indicated concentrations of ketamine (0, 3, and 10 *μ*M) and then cocultured with K562 cells at an effector-to-target cell ratio of 10 : 1. The NKCC was measured by the LDH cytotoxicity assay. KHYG1 cells treated with 10 *μ*M of prednisolone were used as the positive controls. The NKCC data of each of the ketamine groups were similar to that of the control group. ^∗∗∗^*p* < 0.001 vs. prednisolone.

As shown in Figure 1(a), the NKCC was not significantly different between the control and ketamine groups (3 and 10 *μ*M, *p* = 0.61) at all time points (*p* = 0.84) in the *in vitro* experiment (*n* = 24 in each group: 4 h, control: 35.3 ± 5.5%, ketamine 3 *μ*M: 34.3 ± 5.3%, ketamine 10 *μ*M: 34.7 ± 5.3%, 24 h, control: 36.9 ± 5.7%, ketamine 3 *μ*M: 32.8 ± 10.2%, ketamine 10 *μ*M: 38.0 ± 13.6%, 48 h, control: 39.4 ± 12.3%, ketamine 3 *μ*M: 37.0 ± 12.4%, ketamine 10 *μ*M: 32.5 ± 9.1%). The post hoc analysis with the Bonferroni method showed that in the ketamine groups and in the control group, each NKCC value measured at 4 h was similar to those measured at 24 and 48 h when the NKCC was determined at an effector-to-target cell ratio of 20 : 1 (*p* = 1.0, 4 h vs. 24 h and 48 h in all groups, Figure 1(a)). When measured at an effector-to-target cell ratio of 10 : 1 after the 24 h treatment, the NKCC values were also similar between the ketamine and control groups (*n* = 4 in each group, *p* = 0.56, Figure 1(b)).

### 3.2. Analysis of the Combined Data of our Previous Trial with Prostate Cancer Surgery and the Data of the Present Trial with Lung Cancer Surgery

**Figure 2:**
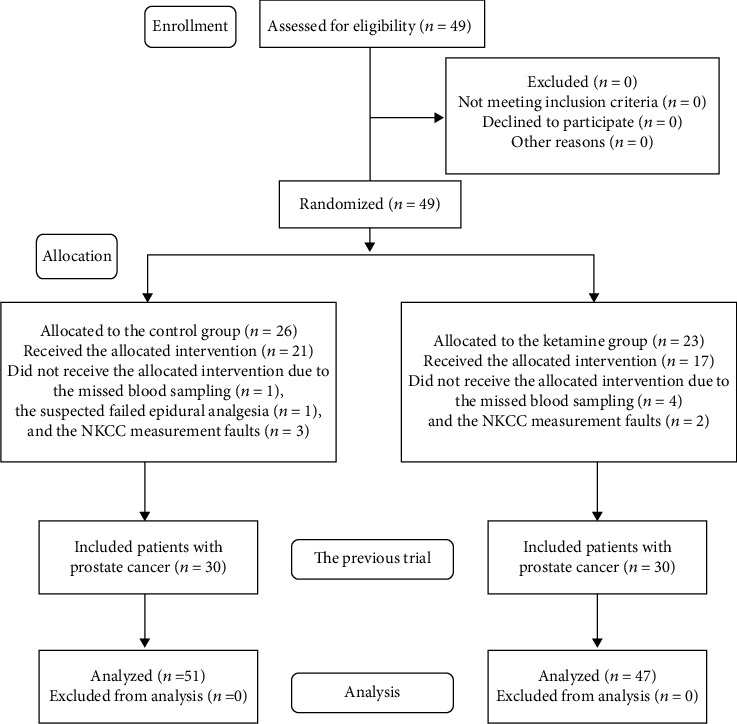
Flow diagram of the patients.

A total of 49 patients who underwent a thoracoscopy-assisted lung lobectomy during the period April 2017–March 2018 at our hospital were enrolled; 38 patients were in the analyses. The data of 60 patients with prostate cancer analyzed in the previous trial were included in the present analyses. We thus analyzed the combined data of 98 patients for the primary and secondary outcomes in the present study. The patients' enrollment profile is illustrated in Figure 2, and their demographics and surgical data details are summarized in Table 1. The clinical data (except for the ketamine dosage) were comparable between the ketamine and control groups. No adverse events or unintended effects associated with this trial were observed.

#### 3.2.1. The NKCC

**Figure 3:**
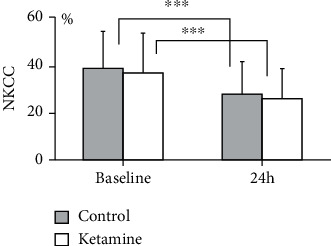
The NKCC in the combined present and previous clinical trials. Blood sampling was performed before the initiation of anesthesia (baseline) and at 24 h after the induction of anesthesia. The NKCC of each patient was measured with a 3.5 hr chromium-51 (^51^Cr) release assay according to the manufacturer's protocol using the reaction of PBMCs and ^51^Cr-labeled K562 cells, at an effector/target ratio of 20 : 1. Compared to each group's baseline value, the NKCC at 24 h in both groups was significantly decreased in almost the same manner (24 h vs. baseline, ^∗∗∗^*p* < 0.001 vs. baseline). There was no significant between-group difference in NKCC of the ketamine and control groups at the baseline (36.6 ± 16.7% vs. 38.5 ± 15.4%, *p* = 0.56) or at 24 h (25.6 ± 12.9% vs. 27.7 ± 13.5%, *p* = 0.49), respectively.

As shown in Figure 3, the NKCC of the patients was not significantly different between the patients who received anesthesia with ketamine and those without ketamine at the baseline (ketamine: 36.6 ± 16.7% vs. control: 38.5 ± 15.4%, *p* = 0.56) and at 24 h (ketamine: 25.6 ± 12.9% vs. control: 27.7 ± 13.5%, *p* = 0.49). Compared to each group's baseline value, the NKCC at 24 h in both groups was significantly decreased in almost the same manner (24 h vs. baseline, *p* < 0.001; Figure 3).

#### 3.2.2. The NLR, the PLR, and the Serum Values of IL-6

**Figure 4:**
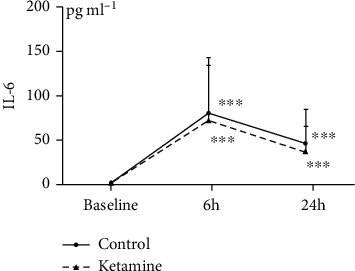
The interleukin- (IL-) 6 values in the combined present and previous trials. Blood sampling was conducted before the initiation of anesthesia (baseline), at 6 h, and at 24 h after the anesthesia induction. A chemiluminescent enzyme immunoassay was used to measure serum IL-6 values according to the manufacturer's protocol. The serum IL-6 levels in the control and ketamine groups changed in essentially the same manner and were similar at each time point. Compared to each group's baseline value, the serum IL-6 values were similarly increased up to a peak value at 6 h (^∗∗∗^*p* < 0.001 vs. baseline) and then decreased slightly, but the significant increase still existed at 24 h (^∗∗∗^*p* < 0.001 vs. baseline). Thus, there was no significant difference in the IL-6 values of the ketamine and control groups at the baseline (1.8 ± 1.2 pg/ml vs. 1.7 ± 1.1 pg/ml, *p* = 0.71), at 6 h (71.4 ± 62.4 pg/ml vs. 80.4 ± 62.6 pg/ml, *p* = 0.50), or at 24 h (36.7 ± 28.8 pg/ml vs. 45.8 ± 38.2 pg/ml, *p* = 0.21), respectively.

We observed no significant between-group difference in the changes in the NLR (*p* = 0.75) or the PLR (*p* = 0.27) (Table 1). The serum IL-6 levels in the ketamine and control groups were similar at each time point (*p* = 0.32) and changed in essentially the same manner (Figure 4); compared to each group's baseline value, the serum IL-6 values in both groups were similarly increased up to a peak value at 6 h (*p* < 0.001 vs. baseline) and then decreased slightly; the significant increase still existed at 24 h (*p* < 0.001 vs. baseline). Thus, there was no significant between-group difference in IL-6 at the baseline (ketamine: 1.8 ± 1.2 pg/ml vs. control: 1.7 ± 1.1 pg/ml, *p* = 0.71), at 6 h (ketamine: 71.4 ± 62.4 pg/ml vs. control: 80.4 ± 62.6 pg/ml, *p* = 0.50), or at 24 h (ketamine: 36.7 ± 28.8 pg/ml vs. control: 45.8 ± 38.2 pg/ml, *p* = 0.21).

## 4. Discussion

We used both basic and clinical approaches to determine whether ketamine modulates NKCC. The results of the *in vitro* experiment demonstrated that the 4, 24, and 48 h ketamine treatment did not change the cytotoxicity of KHYG1 cells. The clinical approach revealed that ketamine does not change the NKCC in the clinical setting as well.

Surgery-induced NK cell dysfunction is thought to participate in some potential mechanisms, e.g., the body's stress response as well as an inflammatory response to surgical trauma. The nociceptive stimuli due to surgical trauma activate the sympathetic nervous system as well as the hypothalamic-pituitary-adrenal axis system. Such a stress response causes immunosuppression in a manner that is corticosteroid-dependent and/or adrenal-dependent [21]. The inflammatory response to surgical trauma, i.e., an acute proinflammatory phase that is followed by a prolonged anti-inflammatory phase, also suppresses NKCC due mainly to the increase in IL-6 [6]. Our starting hypothesis was that ketamine could cancel the surgery-induced immunosuppression since ketamine has antinociceptive and anti-inflammatory effects. However, the results of our clinical trial indicate that this hypothesis is not correct. In contrast, our clinical results demonstrated that ketamine does not change the NKCC in patients undergoing cancer surgery. This study's results are in accord with those of two randomized clinical studies [22, 23] showing that ketamine did not change the NKCC of patients undergoing abdominal [23] and oral maxillofacial [22] surgery.

Another possible factor for postsurgery NK cell dysfunction is the drugs administered (such as anesthetic agents) that directly suppressed anticancer immunity [10, 11, 24]. The results of our *in vitro* investigation indicate that ketamine does not directly suppress NKCC, as this experiment was conducted under well-controlled homogeneous conditions; we investigated the effect of ketamine on NKCC without the complicated biological conditions including surgical stimuli and inflammation. However, two animal studies revealed that nonoperated rats anesthetized with ketamine exhibited reduced NKCC [12, 13] and developed increased lung metastases [12]. Those findings indicate that ketamine does directly reduce NKCC.

The inconsistency between these reported results and our present findings may be due at least in part to the difference in subjects (human vs. rats) as well as in the type of experiments (*in vitro* vs. animal studies). The dose of ketamine used and the treatment time may also be involved. Considering the results of these animal studies, it is of interest that the study by Forget et al. showed that ketamine did depress NKCC in rats without surgery [13]. This result is consistent with the findings reported by Melamed et al. [12]. In contrast, Forget et al. also demonstrated that NKCC in rats administered with ketamine showed a pattern that was similar to that in saline-treated rats when the rats underwent surgery, and the number of lung metastases after surgery was significantly lower in the ketamine-treated rats [13]. These findings in animal models support the clinical results [22, 23], including ours.

This study has some limitations. First, KHYG1 is a human leukemia NK cell line with enhanced NKCC and not a normal human NK cell line. We did not use PBMCs in this study, although PBMCs are thought to include normal NK cells; this is because achieving an adequate number of extracted NK cells for the tests requires a significant amount of whole blood from patients [25]. It is difficult to purify and expand these normal NK cells *in vitro* [26], whereas it is easy to get an adequate number of the cells of the present NK cell lines to test. KHYG1 is the first NK cell line to be widely used to investigate the effects of drugs and natural materials on NKCC, as this cell line is characterized by high purity, which is consistent with the general biological functions of primary NK cells [25, 26]. However, the KHYG1 cells' enhanced cytotoxicity might have affected the present results.

Second, the present clinical trials were conducted at a single institution. In the clinical approach used herein, we analyzed the data of the present clinical trial combined with that of our previous randomized trial so that we had the data of a sufficient number of patients to improve the data's validity. A third study limitation is that we evaluated only NKCC ability, although the NK cell function that is disrupted by surgical stress is not only NKCC but also cytokine production. A reduction of NK cells' cytokine production after surgery has also been reported [6]. Finally, patients undergoing only a minimally invasive surgery were enrolled in our previous trial. A minimally invasive surgery can minimize the patients' surgical trauma, followed by minimized stress and a minimized inflammatory response. In the present trial with lung surgery patients, the neuraxial anesthesia could also have minimized the nociceptive stimuli, followed by a humoral stress response. The minimized nociceptive stimuli and/or inflammatory response in the prostate and lung cancer surgeries may limit the postsurgery NKCC suppression.

In conclusion, although ketamine may be widely considered to suppress NKCC, our basic and clinical approaches to the assessment of the effect of ketamine on NKCC demonstrated that ketamine does not change NKCC. Further studies are warranted to determine ketamine's precise benefit for cancer surgery.

## Figures and Tables

**Table 1:** The characteristics of the patients who underwent lung (lobectomy) or prostate cancer surgery (RARP).

	Control	Ketamine	MD (95% CI)	*p* value
Age (yrs)	68.0 ± 7.1	67.0 ± 7.9	0.9 (−2.0, 3.9)	0.54
Height (cm)	163.2 ± 9.3	163.1 ± 8.9	0.1 (−3.5, 3.7)	0.96
Weight (kg)	63.5 ± 11.0	65.3 ± 11.5	−1.8 (−6.3, 2.69	0.41
Anesthesia (min)	249..8 ± 55.5	257.0 ± 54.4	−7.2 (−28.9, 14.4)	0.51
Surgery (min)	177.2 ± 50.1	183.8 ± 50.7	−6.6 (−26.5, 13.3)	0.51
Propofol (mg)	1231.5 ± 491.2	1334.4 ± 446.6	−103.0 (−288.2, 82.2)	0.27
Remifentanil (*μ*g)	2347.2 ± 734.6	2635.7 ± 926.4	−288.6 (−620.3, 43.1)	0.09
Ketamine (mg)	0	134.0 ± 29.3	−134.0 (−142.4, −125.6)	<0.001
Fentanyl (*μ*g)	265 ± 52	272 ± 47	7 (19, 32)	0.61
Morphine (mg)	10 [10, 10]	10 [10, 10]	NA	0.76
Aceta (mg)	819.2 ± 200.4	836.5 ± 181.3	−17.3 (−92.9, 58.1)	0.65
*Δ*NLR	6.0 [2.3, 15.5]	6.2 [2.6, 14.6]	NA	0.75
*Δ*PLR	−6.4 [−15.4, 12.2]	−3.6 [−12.8, 20.6]	NA	0.27
NRS	0 [0, 3]	0 [0, 2.3]	NA	0.79

*Δ*NLR and *Δ*PLR = value before-after surgery. Aceta: acetaminophen; MD: mean difference; NA: not analyzed; NLR: neutrophil-lymphocyte ratio; NRS: numerical rating scale; PLR: platelet-lymphocyte ratio; RARP: robot-assisted radical prostatectomy.

## Data Availability

Data are available on request. We can make data available on request through the corresponding author, Hidetomo Niwa (email address: niwahide@gmail.com).

## Abbreviations

BIS: Bispectral index
CLEIA: Chemiluminescent enzyme immunoassay
cpm: Counts per minute
Cr: Chromium
EASIA: Enzyme-amplified sensitivity immunoassay
FBS: Fetal bovine serum
IL: Interleukin
LDH: Lactate dehydrogenase
NK: Natural killer
NKCC: Natural killer cell cytotoxicity
NRS: Numerical rating scale.
